# Exposure to common respiratory bacteria alters the airway epithelial response to subsequent viral infection

**DOI:** 10.1186/s12931-016-0382-z

**Published:** 2016-06-03

**Authors:** Carla Bellinghausen, Fahad Gulraiz, Alexandra C. A. Heinzmann, Mieke A. Dentener, Paul H. M. Savelkoul, Emiel F. Wouters, Gernot G. Rohde, Frank R. Stassen

**Affiliations:** Department of Medical Microbiology, School of Nutrition and Translational Research in Metabolism (NUTRIM), Maastricht University, Maastricht, The Netherlands; Department of Respiratory Medicine, Maastricht University Medical Center, Maastricht, The Netherlands; Department of Medical Microbiology & Infection Control, VU University Medical Center, Amsterdam, The Netherlands; P.O. Box 5800, 6202AZ Maastricht, The Netherlands; Department of Cell Biology and Immunology, University of North Texas Health Science Center (UNT Health Science Center), Fort Worth, TX USA

**Keywords:** Bacterial-viral co-infection, Polymicrobial infection, Inflammation

## Abstract

**Background:**

Colonization of the airways with potential pathogenic bacteria is observed in a number of chronic respiratory diseases, such as COPD or cystic fibrosis. Infections with respiratory viruses are known triggers of exacerbations of these diseases. We here investigated if pre-exposure to bacteria alters the response of lung epithelial cells to subsequent viral infection.

**Methods:**

Bronchial epithelial cells (BEAS-2B cells and primary bronchial epithelial cells) were exposed to heat-inactivated *Haemophilus influenzae*, *Pseudomonas aeruginosa* or *Streptococcus pneumoniae* and subsequently infected with respiratory syncytial virus (RSV), type 2 human adenovirus or influenza B. Levels of pro-inflammatory cytokines, viral replication and expression of pattern recognition receptors were determined in culture supernatants and/or cell lysates.

**Results:**

Exposure of BEAS-2B cells to *H. influenzae* before and during RSV-infection synergistically increased the release of IL-6 (increase above calculated additive effect at 72 h: 56 % ± 3 %, mean ± SEM) and IL-8 (53 % ± 12 %). This effect was sustained even when bacteria were washed away before viral infection and was neither associated with enhanced viral replication, nor linked to increased expression of key pattern recognition receptors. *P. aeruginosa* enhanced the release of inflammatory cytokines to a similar extent, yet only if bacteria were also present during viral infection. *S. pneumoniae* did not enhance RSV-induced cytokine release. Surprisingly, adenovirus infection significantly reduced IL-6 release in cells exposed to either of the three tested bacterial strains by on average more than 50 %. Infection with influenza B on the other hand did not affect cytokine production in BEAS-2B cells exposed to the different bacterial strains.

**Conclusion:**

Pre-exposure of epithelial cells to bacteria alters the response to subsequent viral infection depending on the types of pathogen involved. These findings highlight the complexity of microbiome interactions in the airways, possibly contributing to the susceptibility to exacerbations and the natural course of airway diseases.

**Electronic supplementary material:**

The online version of this article (doi:10.1186/s12931-016-0382-z) contains supplementary material, which is available to authorized users.

## Background

Infections of the respiratory tract are a major risk to patients with chronic respiratory diseases, such as chronic obstructive pulmonary diseases (COPD). The majority of acute exacerbations of COPD (AECOPD) is associated with an acute respiratory infection, with viral and/or bacterial pathogens being detected in more than half of all exacerbations [1, 2]. Experimental infections of COPD and asthma patients with human rhinoviruses (HRV) have moreover provided evidence for a causal relationship between an acute infection and the onset of symptoms characteristic for acute exacerbations in both diseases [3, 4]. Other viruses that are detected during AECOPD include respiratory syncytial virus (RSV), influenza viruses and adenovirus strains [5, 6].

Next to viral infection, also bacterial infections can be involved in the development of acute exacerbations. Among the bacterial species that are frequently detected during such episodes are strains of non-typeable (NT) *Haemophilus influenzae*, *Pseudomonas aeruginosa* and *Streptococcus pneumoniae* [1]. Importantly, colonization with these bacteria is also frequently observed in the stable state of the disease. Potential pathogenic microorganisms (PPMs) have been detected in approximately 25 % of COPD patients during stable disease, even when rather insensitive culture-dependent techniques were employed [7–10]. Likewise, increased load of PPMs has also been described for other chronic lung diseases, such as asthma and cystic fibrosis [11–13].

Not only is bacterial colonization associated with an increased risk to develop an acute exacerbation, it is also associated with increased levels of inflammatory markers in the stable state [14–16]. Furthermore, pro-inflammatory cytokines, such as IL-6 and IL-8, have been shown to be elevated in the sputum of frequent exacerbators and during exacerbation [17]. Changes in IL-6 between stable state and exacerbation were found to be particularly pronounced, if the exacerbations were associated with a viral infection [17–19].

AECOPD associated with the detection of a combination of bacterial and viral pathogens have been reported to be particularly severe in terms of inflammation and decline in lung function [20]. Moreover, these events on average required longer hospitalizations [2]. Presence of both, potential pathogenic bacteria and viruses, during the same period of exacerbations have been observed in as much as 12 to 25 % of AECOPD [21, 22]. When specifically looking at AECOPD associated with a positive culture of NT *H. influenzae*, almost half of the patients were also tested positive for at least one respiratory virus [23]. Considering these studies made use of rather insensitive conventional culturing techniques to detect bacteria, these numbers are most likely even underestimating the prevalence of simultaneous presence of bacteria and viruses.

Numerous studies have investigated how primary viral infections increase susceptibility to secondary bacterial infections, for example by disrupting epithelial barriers [24], increasing bacterial attachment or colonization [25–28], or modulating innate and adaptive immune responses [29–33]. On the other hand, consequences of a primary exposure to bacteria on the outcome of secondary viral infections have been less extensively studied [34]. Considering the substantial proportion of patients chronically colonized with bacteria, this concept might - though less well understood - be equally important.

We therefore hypothesized that the response of airway epithelial cells to respiratory viral infections is influenced by pre-exposure to bacterial pathogens. In the present study we investigated how exposure to different respiratory bacteria influences the inflammatory response and susceptibility of respiratory epithelial cells to a secondary infection with respiratory syncytial virus (RSV), influenza B virus and adenovirus.

## Methods

### Cell culture

BEAS-2B cells (ATCC CRL-9609) were cultured in RPMI-1640 (life technologies, Carlsbad, USA) supplemented with 10 % fetal bovine serum (FBS; Lonza, Basel, Switzerland). For experiments, BEAS-2B cells were used for up to 15 passages after cryopreservation (passage number 20 to 35). Culture flasks and multiwell plates were pre-coated with a mixture of bovine collagen (30 μg/ml), human fibronectin (10 μg/ml; both BD Biosciences, San Jose, USA) and bovine serum albumin (BSA, 10 μg/ml, Sigma Aldrich, St Louis, USA). For experiments, BEAS-2B cells were seeded at 10^5^ cells per well in collagen/fibronectin-coated 48-well plates the day before the experiment. Cell layers were approximately 80–90 % confluent at the start of the experiment.

Primary bronchial epithelial cells (pBECs) were kindly provided by the Primary Lung Cell (PLUC) facility Maastricht University Medical Center + (MUMC+, Maastricht, The Netherlands). Lung tissue used for the isolation of pBECs was obtained from the Maastricht Pathology Tissue Collection (MPTC) and originated from tissue resected during lobectomies or pneumonectomies of patients who underwent surgery for lung cancer. Collection, storage and use of tissue and patient data were performed in agreement with the “Code for Proper Secondary Use of Human Tissue in the Netherlands” (http://www.fmwv.nl). The scientific board of the MPTC approved the use of materials for this study under code MPTC2010-019. PBECs were isolated from bronchus rings that were macroscopically free of cancer. Isolation, culture and characterization of cells was performed as previously described [35]. Epithelial character of the cells was confirmed by immunohistochemical staining for cytokeratins 5, 6, 8 and 17. Experiments were performed on cells isolated from seven donors. All donors (median age 67 years, range 55–78 years) were never-smokers or former smokers, and had not been diagnosed with COPD, cystic fibrosis or asthma.

For the seeding of pBECs, the methodology was adapted to account for different generation times of cells derived from different donors and to meet growth requirements of the primary cells. PBECs were seeded at 1.5 × 10^4^ cells per well in pre-coated 24-well plates in B/D medium consisting of 50 % DMEM (life technologies) and 50 % Bronchial Epithelial Basal Medium (BEBM; Lonza), supplemented with bronchial epithelial growth medium (BEGM) singlequots (Lonza), and BSA (1.5 mg/ml, Sigma Aldrich). Experiments were started once cell layers reached approximately 80 % confluence.

### Virus culture

Virus strains were provided by the Dutch National Institute for Public Health and the Environment (RIVM, Bilthoven, the Netherlands).

RSV (strain A2) was propagated in Vero cells and concentrated by precipitation in polyethyleneglycol 6000 as described previously [36]. Influenza B (Yamagata lineage) were propagated in MDCKII cells. Human adenovirus 2 was propagated in HeLa cells.

The infectivity of viruses was quantified by determining the 50 % Tissue Culture Infective Dose (TCID_50_) using the Spearman Karber Method [37].

### Bacterial culture

All bacterial strains used in this study were reference strains obtained from ATCC and were cultured at 37 °C and 5 % CO_2_. NT *H. influenzae* (ATCC 49247) was cultured on Vitox-supplemented chocolate agar plates (Oxoid, Wesel, Germany). *P. aeruginosa* (ATCC 27853) and *S. pneumoniae* (ATCC 49619) were cultured on B/D Columbia blood agar plates (Becton Dickinson, Franklin Lakes, USA).

### Infection protocols

#### Preparation of inactivated bacterial suspensions

Bacterial suspensions were prepared by adding several colonies of an overnight culture to RPMI-1640 medium. These suspensions were heat-inactivated at 65 °C for 1 h. Inactivation was confirmed by plating out aliquots of the suspension on agar plates. Bacteria were then pelleted by centrifugation at 4500 x g for 10 min, washed once with PBS and re-suspended in infection medium. The composition of the infection medium was dependent on the cell type and virus used. Stimulation and infection of BEAS-2B cells with bacteria in combination with RSV and adenovirus was performed in RPMI-1640 supplemented with 2 % FBS (Lonza). For subsequent infection with Influenza B, bacterial suspensions were prepared in serum-free medium consisting of Minimal Essential Medium (life technologies) supplemented with 1 mg/ml proteose peptone, 0.1 mg/ml BSA, 0.2 mg/ml D-glucose monohydrate (all Sigma Aldrich, St Louis, USA) and 0.05 ‰ trypsin/EDTA (life technologies). For experiments on primary cells, infection medium consisted of B/D medium supplemented with BEGM singlequots (both Lonza) except human epidermal growth factor and bovine pituitary extract. The turbidity of the bacterial suspensions was adjusted to 0.5 McFarland (equivalent to approximately 1.5 × 10^8^ cfu/ml).

#### Continuous stimulation

Bacterial suspensions were further diluted 1:10 in infection medium. Cells were first stimulated with bacteria for 4 h, and subsequently infected with the respective virus. For virus infection, culture supernatants were aseptically collected from each well and preserved while cells were exposed for one hour to diluted virus to yield a multiplicity of infection (MOI) of one. After this initial attachment period, cells were washed once with PBS and the original culture medium (including bacteria) was added to back the wells.

#### Pre-exposure protocol

Bacterial suspensions of 0.5 McFarland were prepared as described above. For stimulation of cells, 0.5 ml of the bacterial suspension or control medium were added to each well. After 24 h of exposure, cell layers were washed with PBS and infected with RSV at an MOI of one. Virus particles were allowed to attach for 1 h, after which unbound virus was aspirated, cells layers were washed with PBS and fresh medium was added to the wells.

Cell-free supernatants and cell lysates were collected 24, 48 and 72 h after viral infection and stored at −80 °C until use.

### ELISA

Levels of IL-6 and IL-8 were determined using an enzyme linked immunosorbent assay (ELISA) according to the manufacturer’s instructions (Ready-SET-Go, eBioscience, San Diego, USA). Lower limits of detection were 2.4 pg/ml for IL-6 and 3.2 pg/ml for IL-8.

### Determination of RSV RNA copies

Total RNA from cell lysates was isolated using the FavorPrep Tissue Total RNA purification kit (Favorgen, Pingtung, Taiwan). Remaining genomic DNA was degraded by DNase treatment (Ambion Turbo DNAfree, life technologies). For each sample, 500 ng of total RNA was reverse transcribed using the iScript cDNA synthesis kit (Bio-Rad, Hercules, USA). Real-time PCR was then performed on a MyIQ real-time PCR system (Bio-Rad) using the following primers: Forward 5′- TTTCCACAATATYTAAGTGTCAA-3′; Reverse 5′- TCATCWCCATACTTTTCTGTTA-3′. Viral RNA copy numbers were calculated by using a standard curve derived from in vitro transcribed RSV RNA.

### Statistical analyses

Statistical analysis was performed using Prism 5 (GraphPad, La Jolla, USA) and SPSS 22.0 (IBM, Armonk, USA). Data are presented as mean ± standard error of the mean (SEM) of five to seven independent experiments. For better visualization of the synergistic effects, the additive effect of two pathogens is indicated as a dashed line in some graphs. This theoretical additive effect for a given combination of pathogens was calculated as the sum of cytokine levels of the individual infections. Statistical significance of bacterial and viral challenges, as well as interactions between the two, were determined using a two way analysis of variance (ANOVA) and a Bonferroni post-hoc analysis. To determine statistical significance of bacterial-viral interactions in primary epithelial cells, relative changes over the additive effect were calculated and tested for statistical significance using a one sample *t*-test. Differences in viral RNA copies and infectious particles were tested for statistical significance using a one-way ANOVA. In all cases, effects were considered statistically significant if *p* < 0.05.

## Results

### *H. influenzae* and *P. aeruginosa* synergistically enhance the release of the pro-inflammatory cytokines IL-6 and IL-8 during RSV infection

We first investigated if exposure to respiratory bacterial pathogens can alter the epithelial response to subsequent RSV infection. To this end, we made use of the immortalized bronchial epithelial cell line BEAS-2B, which is frequently used to study airway epithelial responses to pathogens and displays similar expression of pattern recognition receptors (PRRs) as primary bronchial epithelial cells [38]. BEAS-2B cells were stimulated with heat-inactivated preparations of NT *H. influenzae*, *P. aeruginosa* or *S. pneumoniae*. Four hours later, cells were additionally infected with RSV and secretion of IL-6 and IL-8 was monitored over a total period of 72 h.

**Fig. 1:**
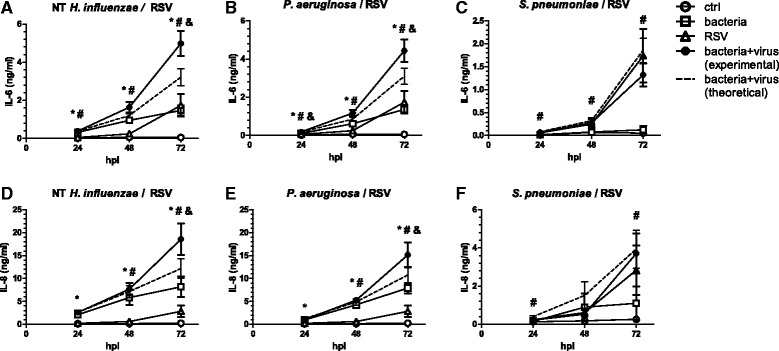
Co-stimulation of epithelial cells with bacteria and virus potentiates inflammatory cytokine release. BEAS-2B cells were first exposed to heat-inactivated preparations of non-typeable (NT) *H. influenzae* (**a**, **d**), *P. aeruginosa* (**b**, **e**) or *S. pneumoniae* (**c**, **f**) for 4 h and subsequently infected with RSV and incubated for up to 72 h in the presence of bacteria. Concentrations of IL-6 (**a–c**) and IL-8 (**d–f**) in culture supernatants were determined by ELISA. The theoretical additive effect (*dashed line*) was calculated as the sum of bacteria and virus-induced cytokine secretion. *Symbols* indicate a statistically significant effect of bacteria alone (*), virus alone (#) and a significant interaction between bacteria and virus (&) as determined by Two Way Repeated Measures ANOVA (*p* < 0.05). hpi: hours post viral infection

Exposure of cells to heat-inactivated *H. influenzae* or *P. aeruginosa* caused significant release of the pro-inflammatory cytokines IL-6 and IL-8, as did infection with RSV alone. When cells were first exposed to heat-inactivated *H. influenzae* (Fig. 1a) or *P. aeruginosa* (Fig. 1b) and subsequently infected with RSV, IL-6 release was synergistically increased: Seventy-two hours after infection, experimentally determined values of IL-6 in the culture supernatants were on average increased by 56 ± 3 % (*H. influenzae*) or 46 ± 9 % (*P. aeruginosa*) compared to the sum of cytokines released by either bacteria or virus alone (indicated as bacteria + virus (theoretical), dashed line). For *S. pneumoniae* on the other hand, there was no significant interaction of bacteria and virus on the induction of IL-6 release at any of the assessed time points (Fig. 1c). Of note, the cytokine response of BEAS-2B cells to heat-inactivated pneumococci was clearly lower than the cells’ response to the other two tested bacterial species and there was no significant effect of *S. pneumoniae* exposure on cytokine release (Fig. 1c, f).

Similar to IL-6, also IL-8 release was synergistically enhanced in cells infected with RSV in the presence of either *H. influenzae* (Fig. 1d) or *P. aeruginosa* (Fig. 1e), whereas we did not detect a significant interaction of *S. pneumoniae* with this virus (Fig. 1f).

RSV and *H. influenzae* also synergistically increased IL-6 release when applied in the reverse order, i.e., when cells were first infected with RSV and subsequently co-stimulated with heat-inactivated bacteria, suggesting interactions between the two pathogens are not exclusive to primary bacterial stimulation (data not shown). However, since we were primarily interested in the effect of a primary bacterial exposure (as a model for bacterial colonization) on secondary viral infections, all results presented here are derived from experiments in which cells were first exposed to bacteria and subsequently infected with virus.

Stimulation with heat-inactivated bacteria and/or RSV did not significantly affect metabolic activity of the cells; differences in cytokine secretion were thus unlikely to be caused by pathogen-specific effects of the treatments on cell viability (Additional file 1: Figure S1A).

Due to the limited possibilities for bacterial control or clearance in our in vitro setting, inactivating the bacterial suspension was inevitable to avoid bacterial overgrowth of the epithelial cultures during the 3 day experimental period. To exclude any effect of the mode of inactivation on our readouts, we repeated the experiments using a low dose of gentamicin (4 μg/ml) to inhibit growth of *H. influenzae,* instead of heat-inactivating the bacteria. Although the results failed to meet statistical significance, we still observed a trend towards a synergistic interaction between *H. influenzae* and RSV on IL-6 release (mean increase 65 % ± 39 %, *p* = 0.065; Additional file 1: Figure S1B).

### Pre-exposure of cells to heat-inactivated *H. influenzae* is sufficient to synergistically increase RSV-induced cytokine release

Several respiratory viruses, including RSV, have previously been shown to enhance attachment of a variety of bacterial species to cells of the respiratory tract [27, 28, 39–41]. Due to the prolonged incubation during which the cells are exposed to both pathogens (bacteria and viruses), it cannot be excluded that viral infection enhances bacterial binding also in our experimental setting. Such augmented bacterial attachment and concomitant enhanced recognition might also explain an increased inflammatory response. In a second set of experiments, we therefore investigated if the altered inflammatory response of co-stimulated cells required the presence of bacteria during viral infection.

**Fig. 2:**
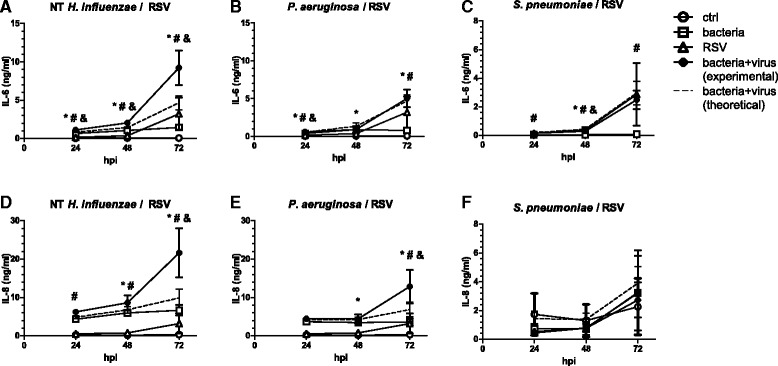
Pre-exposure of cells to NT *H. influenzae* increases production of inflammatory cytokines upon RSV infection. BEAS-2B cells were first exposed to heat-inactivated preparations of non-typeable (NT) *H. influenzae* (**a**, **d**), *P. aeruginosa* (**b**, **e**) or *S. pneumoniae* (**c**, **f**) for 24 h, after which bacteria were washed away and cells were infected with RSV for up to 72 h. Concentrations of IL-6 (**a–c**) and IL-8 (**d–f**) in culture supernatants were determined by ELISA. The theoretical additive effect (*dashed line*) was calculated as the sum of bacteria and virus-induced cytokine secretion. *Symbols* indicate a statistically significant effect of bacteria alone (*), virus alone (#) and a significant interaction between bacteria and virus (&) as determined by Two Way Repeated Measures ANOVA (*p* < 0.05). hpi: hours post viral infection

Cells were pre-exposed to bacteria for 24 h, after which cell layers were washed thoroughly with PBS and infected with RSV. This pre-exposure of cells to *H. influenzae* was sufficient to synergistically increase RSV-induced IL-6 release at all assessed time points. Pre-exposure also increased IL-8 release after subsequent infection with RSV, with the interaction reaching statistical significance at 72 h after infection and a trend towards statistical significance at the earlier time points (24 h: *p* = 0.077; 48 h: *p* = 0.089; Fig. 2d).

In cells pre-exposed to *P. aeruginosa* before RSV-infection, we only detected a small, yet statistically significant synergistic effect on IL-6 production 24 h after infection (mean increase over additive effect 8 % ± 2 %, *p* = 0.002), but not at the later time points (Fig. 2b). IL-8 secretion in response to RSV, however, was still synergistically enhanced after pre-exposure of cells to *P. aeruginosa* at 72 h after infection (Fig. 2e). In line with the results obtained from a continuous exposure to bacteria, pre-exposure of cells to heat-inactivated preparations of *S. pneumoniae* did not significantly affect cytokine release after RSV infection (Fig. 2 c and f). As for the continuous exposure protocol, also during the pre-exposure protocol there was no evidence for a significant effect of the treatments on cell viability (Additional file 1: Figure S1C).

Taken together, these results indicate that pre-exposure of cells with NT *H. influenzae,* is sufficient to potentiate RSV-induced cytokine release and that continuous presence of bacteria is not required. The increased inflammatory response of cells pre-exposed to NT *H. influenzae* was thus unlikely to be triggered by enhanced bacterial attachment to the cells.

### Influence of previous exposure to bacteria on RSV-induced IL-6 production by primary bronchial epithelial cells

We next sought to confirm our findings in primary bronchial epithelial cells (pBECs). Therefore, bronchial epithelial cells were isolated from seven non-smoking individuals without chronic respiratory diseases such as COPD, asthma or cystic fibrosis (median age 67 years; range 55–78 years). Presence of chronic respiratory diseases was excluded based on medical history, pulmonary function tests and imaging. The cells were treated with suspensions of heat-inactivated bacteria and subsequently infected with RSV according to the pre-exposure protocol described in the Methods section of this article.

Cytokine release, particularly in response to RSV infection, varied considerably between cells from different donors (Table 1).

**Table 1:** IL-6 release by pBECs pre-exposed to heat-inactivated bacteria and subsequently infected with RSV

	24 hpi		48 hpi		72 hpi	
	- RSV	+ RSV	- RSV	+ RSV	-RSV	+ RSV
ctrl	6.1 ± 1.1	179.1 ± 77.2	8.4 ± 2.6	208.5 ± 83.3	6.6 ± 1.8	234.3 ± 91.6
+hiNTHI	8.4 ± 1.6	206.8 ± 88.0	11.4 ± 2.6	258.7 ± 116.3	10.3 ± 2.7	324.2 ± 147.3
+hiPA	19.5 ± 14.9	225.0 ± 123.0	21.4 ± 16.3	288.7 ± 120.6	18.6 ± 13.9	335.3 ± 139.7
+hiSP	4.5 ± 0.9	177.8 ± 74.8	6.0 ± 1.7	210.0 ± 88.0	6.2 ± 1.6	238.7 ± 100.1

Values are given in pg/ml and represent the mean ± SEM (*n* = 7). hpi: hours post (viral) infection, *hi* heat-inactivated, *NTHI* non-typeable *H. influenzae*, *PA P. aeruginosa*, *SP S. pneumoniae, RSV* respiratory syncytial virus

**Fig. 3:**
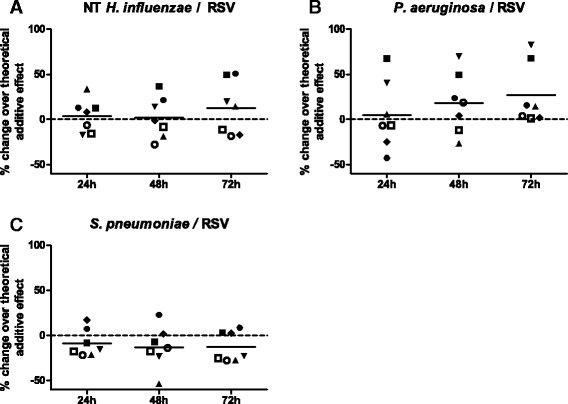
Exposure of pBECs to NT *H. influenzae* or *P. aeruginosa* enhances IL-6 release. PBECS of seven donors were pre-exposed to heat-inactivated preparations of non-typeable (NT) *H. influenzae* (**a**)*, P. aeruginosa* (**b**)*,* or *S. pneumoniae* (**c**) for 24 h, after which bacteria were washed away and cells were infected with RSV for up to 72 h. IL-6 levels in culture supernatants 24, 48 and 72 h after viral infection were determined by ELISA. Due to considerable inter-individual variation in absolute cytokine levels, data are shown as relative changes of experimentally determined cytokine concentrations over the theoretical additive effect for each donor. Results obtained using cells from each donor are denoted with the same symbol in all datasets. hpi: hours post viral infection

In order to account for inter-individual variability, results in Fig. 3 are expressed as percent changes relative to a calculated additive effect (i.e., relative to the sum of cytokine concentrations released in response to each pathogen alone) for each donor. Pre-exposure to *H. influenzae* increased RSV-induced IL-6 release by cells from four out of seven donors above this additive effect (Fig. 3a). Likewise, pre-exposure to *P. aeruginosa* synergistically enhanced the IL-6 release following RSV infection in four out of seven donors, with a trend towards statistical significance 72 h after infection (*p* = 0.09, Fig. 3b). Moreover, and also in line with the results obtained in BEAS-2B cells, *S. pneumoniae* did not further increase IL-6 release after RSV-infection in pBECs (Fig. 3c). In contrast, however, pre-exposure of pBECs to either bacterial species did not significantly affect virus-induced IL-8 secretion (data not shown).

Up to 48 h after viral infection, there was no effect of the treatments on metabolic activity of pBECs; Seventy-two hours after infection, cell viability was modestly decreased in virus-infected cells, yet there were no differences between cells pre-treated with the different bacteria that would explain altered cytokine secretion (Additional file 1: Figure S1D).

### Enhanced release of inflammatory mediators is not linked to enhanced viral replication

**Fig. 4:**
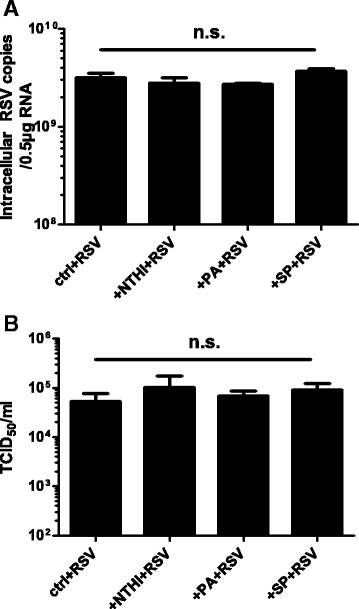
Pre-exposure of cells to bacteria has no significant effect on RSV replication. BEAS-2B cells were exposed to heat-inactivated bacteria for 24 h, after which cell layers were washed and infected with RSV for another 72 h. **a** Intracellular RSV RNA copies were determined by real-time PCR. **b** Release of infectious progeny in culture supernatants was measured by determining the TCID_50_. NTHI: non-typeable *H. influenzae*, PA: *P. aeruginosa*, SP: *S. pneumoniae*, n.s.: not significant

Exposure to bacterial pathogens has previously been suggested to enhance viral attachment and replication in lung epithelial cells [36, 42]. We therefore tested if the enhanced inflammatory response of BEAS-2B cells towards RSV after pre-exposure with bacteria was due to enhanced viral replication. Neither intracellular viral RNA levels (Fig. 4a) nor the release of infectious progeny (Fig. 4b) was significantly altered when cells were pre-exposed to either of the three bacteria used in this study. Nevertheless, viral replication seemed at least indirectly to be required to potentiate cytokine release, since *H. influenzae* in combination with UV-inactivated virus did not cause increased secretion of IL-6 (Additional file 1: Figure S1E). In agreement with this, synergy between the pathogens was most pronounced after cells mounted a cytokine response against the virus itself, i.e., at 48 h after viral infection and later.

To test if exaggerated inflammation was linked to increased expression of key PRRs involved in the recognition of bacterial and/or viral structures, we next measured mRNA levels of TLR2, TLR3, TLR4 and RIG-I. However, there was no significant interaction between bacteria and viruses on the expression levels of any of these genes (Additional file 1: Figure S2).

### Changes in the virus-induced inflammatory response after bacterial pre-exposure are species-specific

Because of the pathogen-specificity we observed when investigating different bacteria in combination with RSV, we next evaluated the inflammatory response of bacterially challenged epithelial cells during infection with different viruses. Next to RSV, rhinoviruses, influenza viruses and specific adenoviruses are among the most frequently detected respiratory viral pathogens [5, 43]. Since the influence of bacterial exposure on infection with human rhinovirus has already been the subject of previous studies [36, 42], we now assessed combinations of different bacteria with influenza B virus and a type 2 human adenovirus.

**Fig. 5:**
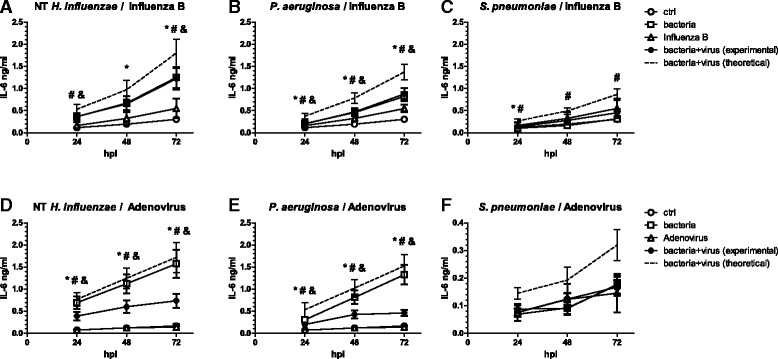
Co-stimulation of cells with bacteria and viruses influences cytokine release in a pathogen-specific manner. BEAS-2B cells were first pre-exposed to either NT *H. influenzae* (**a**, **d**), *P. aeruginosa* (**b**, **e**) or *S. pneumoniae* (**c**, **f**) for 4 h and subsequently infected with Influenza B (**a–c**) or Adenovirus (**d–f**) for up to 72 h in the presence of bacteria. Concentrations of IL-6 in culture supernatants were determined by ELISA. The theoretical additive effect (*dashed line*) was calculated as the sum of bacteria and virus-induced cytokine secretion. *Symbols* indicate a statistically significant effect of bacteria alone (*), virus alone (#) and a significant interaction between bacteria and virus (&) as determined by Two Way Repeated Measures ANOVA (*p* < 0.05). hpi: hours post viral infection

In our hands, infection with influenza B virus only induced modest IL-6 release (Fig. 5 a–c). Although we found a statistically significant interaction between *H. influenzae* and influenza B virus (measured cytokine levels lower than the calculated additive effect 24 and 72 h after infection), as well as between *P. aeruginosa* and influenza B (measured cytokine levels lower than the calculated additive effect at all time points), co-stimulated cells still released cytokines at levels comparable to those of cells stimulated with bacteria only.

Human adenovirus on the other hand did not induce IL-6 production by itself, but significantly reduced cytokine release induced by the different bacteria. When compared to the additive effect of the pathogens, stimulation of cells with adenovirus after bacterial exposure reduced IL-6 release at 72 h by 57 % ± 6 % in combination with NT *H. influenzae* (Fig. 5d) and 68 % ± 4 % in combination with *P. aeruginosa* (Fig. 5e). For IL-8, there was a significant interaction between all three bacteria in combination with human adenovirus 72 h after viral infection. Measured IL-8 levels were on average 39 % ± 9 % (for NT *H. influenzae*), 42 % ± 4 % (for *P. aeruginosa*) and 83 % ± 2 % (for *S. pneumoniae*) below the additive effect of these pathogens.

In summary, the effect of subsequent challenges with bacteria and viruses on release of inflammatory mediators was not only dependent on the bacterial species used, but also virus-dependent.

## Discussion

*H. influenzae* is the most commonly found PPM in the lower respiratory tract of COPD patients. We here demonstrate that exposure of the bronchial epithelial cell line BEAS-2B to heat-inactivated *H. influenzae* significantly augments RSV-induced release of the pro-inflammatory cytokines IL-6 and IL-8. *H. influenzae* has previously been described to enhance the secretion of IL-8 in response to certain strains of another major respiratory virus, human rhinovirus (HRV), by up-regulating expression of TLR3 [42]. In our experimental model of bacterial exposure and RSV-infection, the synergy between heat-inactivated *H. influenzae* and RSV on the release of pro-inflammatory cytokines was not reflected in the expression levels of TLR3, or other pattern recognition receptors (PRRs) relevant for the detection of RSV and/or *H. influenzae* [44–47].

Increased bacterial or viral binding is considered a major feature in the pathogenesis of polymicrobial infections. RSV infection has previously been described to promote fimbriae-mediated attachment of *H. influenzae* to epithelial cells [41, 48]. However, considering that the pro-inflammatory effects of pre-exposure to heat-inactivated *H. influenzae* were sustained even when unbound bacteria were washed away prior to RSV infection, the increased release of IL-6 and IL-8 is most likely not a consequence of greater bacterial binding. Conversely, exposure of lung epithelial cells to *H. influenzae* has been shown to increase the cells’ susceptibility to secondary infection with major group HRV by upregulating their cellular entry receptor, ICAM-1 [36, 42]. Although neutralizing antibodies for ICAM-1 can reduce RSV infection in vitro, it is not the main functional receptor for this virus [49, 50]. Correspondingly, the exaggerated inflammation we observed in our model was not linked to increased viral endpoint titers nor increased levels of intracellular viral RNA, suggesting alterations in intra- or intercellular signaling rather than a generalized increase in viral replication as a cause. Nevertheless, viral replication was at least indirectly required, since UV-inactivated virus did not further increase the secretion of inflammatory cytokines in our experimental model. Interestingly, treatment of epithelial cells with heat-killed *H. influenzae* can reduce bacterial and fungal burden after secondary infection with several pathogens, and also limit influenza virus replication in a mouse model of influenza virus pneumonia [51, 52]. Effectiveness against other viruses has yet to be evaluated, but was not evident for RSV in our model.

Similar to *H. influenzae*, also *P. aeruginosa* elicited a strong increase in the secretion of pro-inflammatory cytokines IL-6 and IL-8, and like *H. influenzae*, potentiated RSV-induced cytokine release. However, in BEAS-2B cells, continuous presence of *P. aeruginosa* during viral infection was required to observe a sustained synergistic effect on IL-6 release. *P. aeruginosa* and RSV have been described to interact directly and RSV has hence been suggested as a coupling agent between bacteria and cells, thus enhancing bacterial attachment [53]. Such an interaction might explain the diverging results we obtained using different exposure protocols.

The third bacterial pathogen we investigated in this study was *S. pneumoniae*, a Gram-positive opportunistic pathogen of the respiratory tract. In our experimental setting, heat-inactivated preparations of *S. pneumoniae* did not increase secretion of IL-6 or IL-8. This lack of an epithelial reaction towards *S. pneumoniae* might be attributable to the fact that we used heat-inactivated preparations of bacteria, preventing the expression of important virulence factors. Also, we did not observe a consistent significant interaction between *S. pneumoniae* and RSV in our model in terms of eliciting an inflammatory response. Importantly, RSV has recently been found to directly interact with *S. pneumoniae* [54]*.* Bacterial binding to the viral G protein triggered profound alterations in the bacterial transcriptome and lead to increased expression of a set of virulence factors. Unfortunately, in our experimental setting inactivation of bacteria was inevitable to prevent bacterial overgrowth and synergistic effects of *S. pneumoniae* and RSV might therefore be obscured. Altered bacterial virulence due to direct interactions between pathogens adds another layer of complexity to the study of polymicrobial infections.

Our experiments in pBECs revealed considerable inter-individual differences between cells from different donors, not only in terms of absolute cytokine concentrations, but also with respect to the effect of bacterial exposure on subsequent infection. Although we aimed to include pBEC donors of a relatively homogenous group in terms of age, smoking-status and the absence of COPD, asthma and cystic fibrosis as underlying pulmonary diseases, differences that arise due to their clinical background or the isolation procedure cannot fully be excluded as a source of variability. Notably, cytokine release was evidently lower in primary cells than in BEAS-2B cells, particularly in response to bacteria. This less responsive phenotype might contribute to the differences we observed between primary cells and the immortalized cell line. Nonetheless, at 72hpi, results obtained with cells from four out of seven donors were in line with the data obtained in BEAS-2B cells, showing a more than additive effect of pre-exposure with *H. influenzae* or *P. aeruginosa* and RSV infection.

The effect of an exposure to bacteria prior to viral infection on cellular inflammatory responses was not only dependent on the bacterial species, but was also virus-specific. Cells exposed to bacteria and subsequently infected with influenza B still released cytokine levels comparable to those of cells exposed to bacteria alone. Even though secondary bacterial infection is a major contributor to influenza virus pathology the reverse order of sequential infections does not seem to have such detrimental effects [34]. This is in line with previous data by Lee et al., who demonstrated a lack of effect on the course of the disease when mice were exposed to *H. influenzae* type b before an influenza challenge, despite a strong lethal synergism when mice were infected in the reverse order [55]. Application of *H. influenzae* lysates can even confer a temporary protection against subsequent influenza infection by boosting local innate responses [52].

In contrast, cytokine levels produced by cells infected with adenovirus after an initial bacterial exposure were significantly reduced. Decreased levels of IL-6 and IL-8 after exposure to bacterial stimuli in combination with adenovirus might be attributed to the adenoviral proteins E1A and E1B, which have previously been reported to reduce the induction of IL-6 in response to bacterial and inflammatory stimuli in human epithelial cells [56, 57].

Using an in vitro model of bronchial epithelial cells allowed us to investigate pathogen specific interactions in a controlled environment. Being the first tissue to be exposed to infectious particles, the airway epithelium is crucial for initiating a first immune response. However, the possibilities to mimic a chronic exposure to bacteria, as would be the case during a bacterial colonization of the lungs, are limited in vitro. Therefore, more studies are needed to examine, for example, the impact of immune cells and the development of tolerance in this context. Extrapolating biological significance from the magnitude of an effect observed in vitro may be difficult. In a model of bacterial co-colonization, Ratner et al. showed that interactions of pathogens on the release of cytokines in vitro which were in a similar order of magnitude as the ones we observed in our study, could indeed translate to a biologically relevant effect in vivo [58].

Even though culture-dependent techniques usually only revealed a limited number of bacterial genera in the lower respiratory tract of colonized individuals, molecular techniques suggest that the lung microbiota is in fact far more complex. Furthermore, changes in the lung microbiome in asthma and COPD have been reported in several studies [59–61]; particularly pathogenic proteobacteria, such as *H. influenzae*, were more frequently found in the lungs of patients suffering from these diseases than in control subjects. It can be assumed that not only the presence of certain bacterial species, but also the composition of the local microbiota in the respiratory tract affects the response towards viral pathogens. In view of the pathogen-specific effects we observed in our study, it would therefore be interesting to expand this concept further towards a more complex representation of the lung microbiome in health and disease.

## Conclusions

We show that pre-exposure of airway epithelial cells to heat-inactivated NT *H. influenzae* and *P. aeruginosa* aggravates the production of pro-inflammatory cytokines in response to subsequent infection with RSV, but not influenza B virus or adenovirus. Taken together, our data provide experimental evidence for the pleiotropic effects of microbial interactions on pulmonary inflammation. If these findings can be translated into the clinical setting, it might enable clinicians to identify patients at high risk for developing more severe viral infections on the basis of their bacteriological status.

## Additional file

Additional file 1: Supplemental information. (ZIP 83 kb)
