# Supplementary material for: Exposure to common respiratory bacteria alters the airway epithelial response to subsequent viral infection
Source: Respir Res. 2016 Jun 3;17:68. doi: 10.1186/s12931-016-0382-z (PMC4891894; doi:10.1186/s12931-016-0382-z)
Supplement: Additional file 1: — Supplemental information. (ZIP 83 kb) [file 12931_2016_382_MOESM1_ESM.zip › 20160419 Supporting information Revision.docx]

**Supplemental information**

***Methods:***

***Stimulation of BEAS-2B cells with H. influenzae and RSV in the presence of gentamicin.*** Suspensions of NT *H. influenzae* (ATCC 49247), were adjusted to a turbidity of 0.5 McFarland and further diluted 1:10 in RPMI-1640 (life technologies) supplemented with 4 µg/ml gentamicin (centrafarm, Etten-Leur, The Netherlands) and 2% FBS (Lonza). Stimulation and infection with RSV was further carried out as described in the protocol for continuous stimulation in the methods section of the main part of this manuscript.

***Determination of metabolic activity.***

As and indicator for cell viability, metabolic activity of the cells was measured by means of an Methylthiazolyldiphenyl-tetrazolium bromide (MTT) assay. BEAS-2B cells and primary bronchial epithelial cells (pBECs) from three donors were exposed to bacteria and/or virus according to the treatment protocols described in the Materials and Methods section of the main article. Seventy-two hours after viral infection, the medium was aspirated and replaced with infection medium containing 0.5 mg/ml MTT (Sigma Aldrich, St Louis, USA). After incubating the cells for 1h at 37 °C / 5% CO_2_ cells, culture supernatants were discarded and colored formazan crystals were dissolved in equal volumes of dimethyl sulfoxide (DMSO). Absorption was measured at 540nm and metabolic activity was calculated relative to mock treated cells as $\frac{OD540(treated)}{OD540(control)} \times100\%$.

***UV-inactivation of virus.***

To inactivate RSV preparations, PEG-concentrated RSV pool was UV-irradiated on ice for 30 minutes under UV light. Loss of infectivity was confirmed by exposing Vero cells to inactivated virus and monitoring for cytopathic effects (CPE). Inactivation was considered successful, if cells did not develop CPE after 7d of exposure.

***Real-time PCR.***

RNA isolation, preparation of cDNA and real-time PCR was performed as described in the Methods section of this article, the primer sequences used for amplification can be found in Table S1. Results are expressed as fold changes and were calculated using the 2^-ΔΔCt^ method (1), using β-actin as internal control.

**Table S1.**

| **gene product** | **primer sequence** |
| --- | --- |
| TLR2 | Forward: 5’-GGCCAGCAAATTACCTGTGTG-3’  Reverse: 5’-AGGCGGACATCCTGAACCT-3’ |
| TLR3 | Forward: 5’-CCTGGTTTGTTAATTGGATTAACGA-3’  Reverse: 5’-TGAGGTGGAGTGTTGCAAAGG-3’ |
| TLR4 | Forward: 5’- CTGCAATGGATCAAGGACCA -3’  Reverse: 5’- TTATCTGAAGGTGTTGCACATTCC-3’ |
| RIG-I | Forward: 5’-ACTCTGGTTGCCAAATCCAC- 3’  Reverse: 5’-TGGAAGGGATCAGCAAAGAG-3’ |
| β-actin | Forward: 5’-TGGAGAAAATCTGGCACCAC-3’  Reverse: 5’-GAGGCGTACAGGGATAGCAC- 3’ |

***Supplemental figures***

***Figure legends***

**Figure S1**. ***(A)*** Metabolic activity of BEAS-2B cells exposed to heat-inactivated (hi) bacteria and/or RSV according to the continuous exposure protocol described in Materials and Methods (MTT assay 72h after viral infection) ***(B)*** IL-6 release by BEAS-2B cells stimulated with NT *H. influenzae* (NTHI) and subsequently infected with RSV for 72h in the presence of gentamicin. Symbols indicate a statistically significant effect of bacteria (*) or virus (#) as determined by Two Way Repeated Measures ANOVA (p<0.05). ***(C)*** Metabolic activity of BEAS-2B cells exposed to hi bacteria and/or RSV according to the pre-exposure protocol in materials and methods (MTT assay 72h after viral infection). ***(D)*** Metabolic activity of pBECs pre-exposed to hi bacteria and subsequently infected with RSV (MTT assay 72h after viral infection) ***(E)*** IL-6 release by BEAS-2B cells stimulated with hi-NTHI and subsequently exposed to UV-inactivated preparations of RSV (UV-RSV) for 72h. Graphs show mean ± SEM of at least three independent experiments. NTHI: non-typeable *H. influenzae*, PA: *P. aeruginosa*, SP: *S. pneumoniae*, RSV: Respiratory Syncytial Virus.

**Figure S2**. Expression of pattern recognition receptors after priming of cells with heat-inactivated NT *H. influenzae* and subsequent infection with RSV (72hpi). Expression levels of TLR2 ***(A)***, TLR3 ***(B)***, TLR4 ***(C)*** and RIG-I ***(D)*** were determined by RT real-time PCR and are expressed as fold change over sham-treated controls. Graphs show mean ± SEM of at least four independent experiments.

***Supplemental Reference***

1. **Livak KJ and Schmittgen TD.** Analysis of relative gene expression data using real-time quantitative PCR and the 2(-Delta Delta C(T)) Method. *Methods* 25: 402-408, 2001.
