# Supplementary figures and images for: Exposure to common respiratory bacteria alters the airway epithelial response to subsequent viral infection
Source: Respir Res. 2016 Jun 3;17:68. doi: 10.1186/s12931-016-0382-z (PMC4891894; doi:10.1186/s12931-016-0382-z)

# S2

## A.

### TLR2

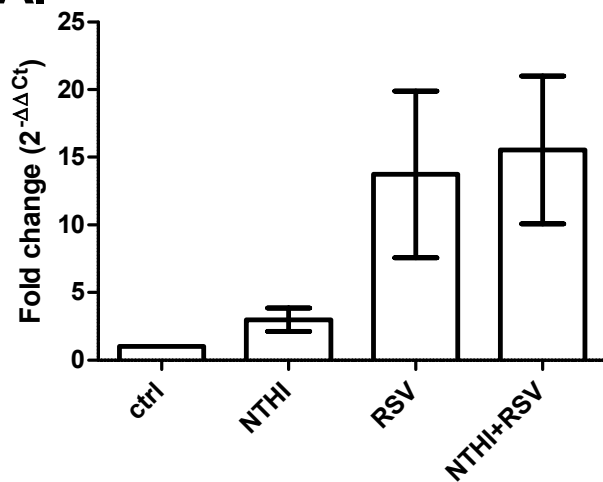

## B.

### TLR3

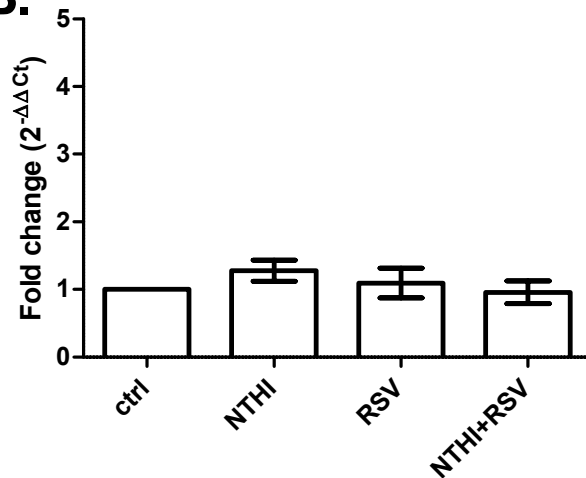

## C.

### TLR4

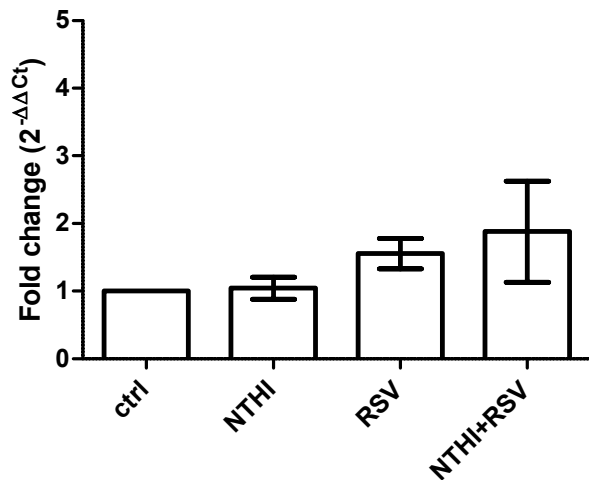

## D.

### RIG-I

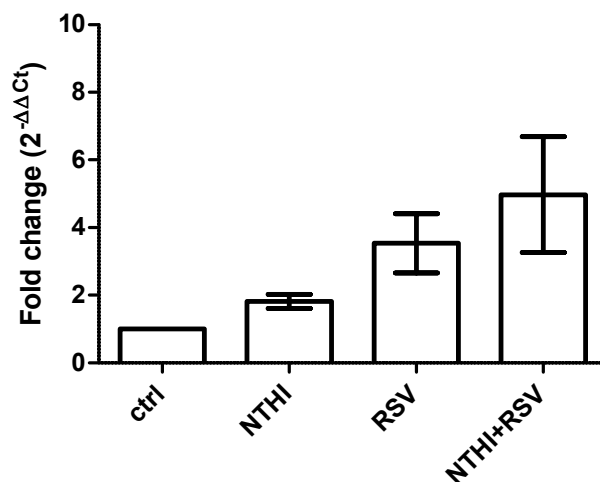

Supplement: Additional file 1: — Supplemental information. (ZIP 83 kb) [file 12931_2016_382_MOESM1_ESM.zip › Supp Fig 2.pdf]

# Supplemental Figures S1

**A.**

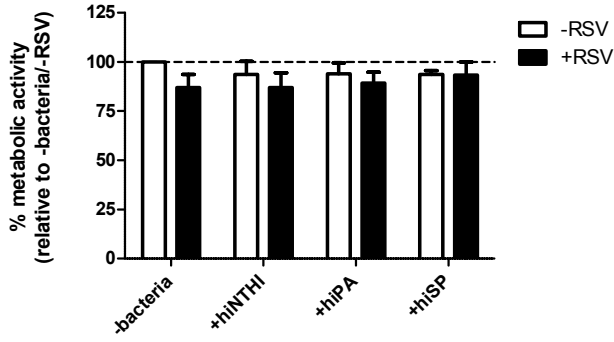

**B.**

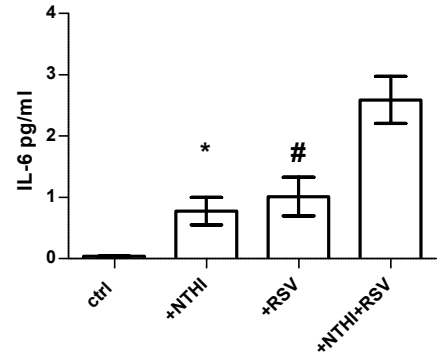

**C.**

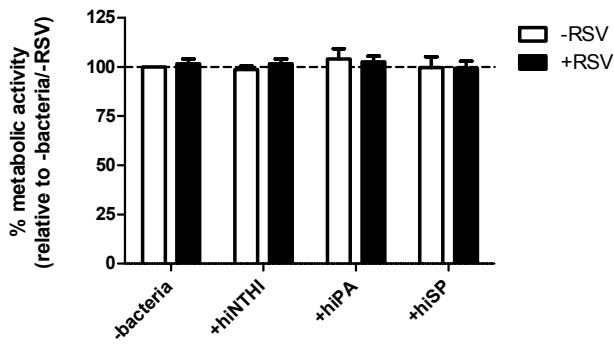

**D.**

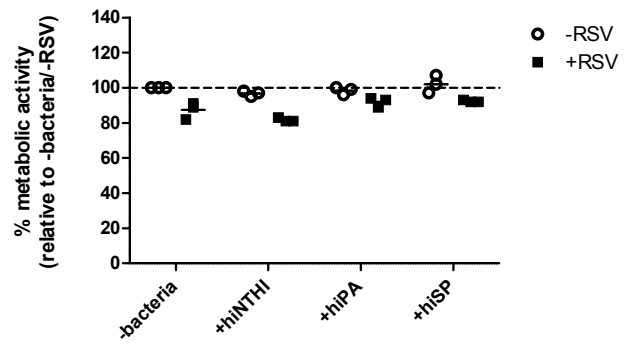

**E.**

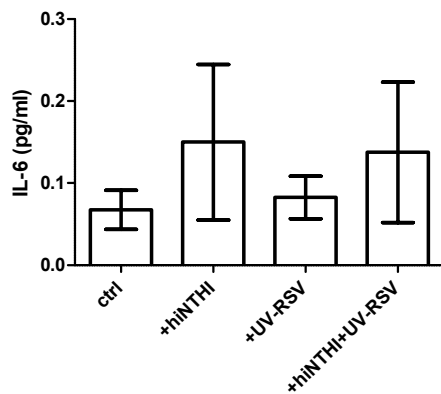

Supplement: Additional file 1: — Supplemental information. (ZIP 83 kb) [file 12931_2016_382_MOESM1_ESM.zip › Supp Fig 1 (Revised).pdf]
